# Characterising Pre-pubertal Resistance to Death from Endotoxemia

**DOI:** 10.1038/s41598-017-16743-1

**Published:** 2017-11-29

**Authors:** Rose Joachim, Freeman Suber, Lester Kobzik

**Affiliations:** Harvard T. H. Chan School of Public Health, Boston, MA USA

## Abstract

Sepsis is a common and deadly syndrome in which a dysregulated host response to infection causes organ failure and death. The current lack of treatment options suggests that a new approach to studying sepsis is needed. Pre-pubertal children show a relative resistance to death from severe infections and sepsis. To explore this phenomenon experimentally, we used an endotoxemia model of sepsis in mice. Following intra-peritoneal injection of endotoxin, pre-pubertal mice showed greater survival than post-pubertal mice (76.3% vs. 28.6%), despite exhibiting a similar degree of inflammation after two hours. Age-associated differences in the inflammatory response only became evident at twenty hours, when post-pubertal mice showed prolonged elevation of serum cytokines and differential recruitment of peritoneal immune cells. Mechanistically, prevention of puberty by hormonal blockade or acceleration of puberty by oestrogen treatment led to increased or decreased survival from endotoxemia, respectively. Additionally, the adoptive transfer of pre-pubertal peritoneal cells improved the survival of post-pubertal recipient mice, while post-pubertal peritoneal cells or vehicle did not. These data establish a model for studying childhood resistance to mortality from endotoxemia, demonstrate that oestrogen is responsible for an increased susceptibility to mortality after puberty, and identify peritoneal cells as mediators of pre-pubertal resistance.

## Introduction

Despite decades of research, sepsis remains a major cause of death in the United States and throughout the world. Sepsis is a complex syndrome in which the host response to infection becomes dysregulated, causing life-threatening organ dysfunction and failure despite appropriate antibiotic treatment and supportive care^1^. Currently, there are no effective drugs among those currently approved to treat the sepsis syndrome. However, this regrettable lack of therapeutic options is not due to a lack of effort. Over 100 Phase II and Phase III clinical trials for drugs targeting sepsis have been carried out since 1979 and have failed^2^. The difficulty in developing drugs to treat sepsis reflects the extreme complexity and variability of the condition. Despite the attempted division of sepsis pathophysiology into early hyper-inflammatory and later hypo-inflammatory phases, actual patients fall within a wide spectrum between these two polarities^3^. The effort to develop new, targeted therapies for sepsis could benefit from a deepened understanding of the factors that promote progression to mortality versus resolution and a return to homeostasis.

To better understand the regulatory systems that determine severity and outcomes in sepsis, it would be useful to study a population that exhibits a natural resistance to sepsis mortality. Intriguingly, epidemiologic studies point to human children as a demographic that meets this criterion. Compared to adults, the paediatric population shows decreased mortality from a variety of conditions that typically involve immune system dysregulation as part of the pathophysiology. In studies that offer detailed analysis by age, this difference in mortality is particularly apparent in a specific subset of paediatric patients: pre-pubertal children. For example in mortality data from the 1918 pandemic flu, children between the ages 5 and 14 (age of puberty onset then) showed decreased morbidity and mortality in comparison to young adults, despite equal rates of infection^4,5^. Similar data exist for mortality from other severe infections such as the 1957 pandemic flu^6^, tuberculosis^4,7^, Ebola^8,9^, yellow fever^10^, pneumonia^11^, chicken pox^12,13^, and several studies specifically focused on sepsis^14–16^.

In this work, we established a simple mouse model of pre-pubertal resistance to sepsis using intra-peritoneal injection of endotoxin. Endotoxin, or more specifically, lipopolysaccharide (LPS), is a component of the gram-negative bacterial cell wall and rapidly initiates an intense inflammatory response via activation of toll-like receptor 4. We used this model to characterise the immune responses of the pre- and post-pubertal age groups as well as to explore possible mechanisms driving the differences between them. By studying the mechanisms that drive pre-pubertal resilience to mortality, we hope to elucidate the adult-specific changes in the immune response that begin during the pubertal transition.

## Results

### Pre-pubertal mice exhibit resistance to mortality from endotoxemia

**Figure 1:**
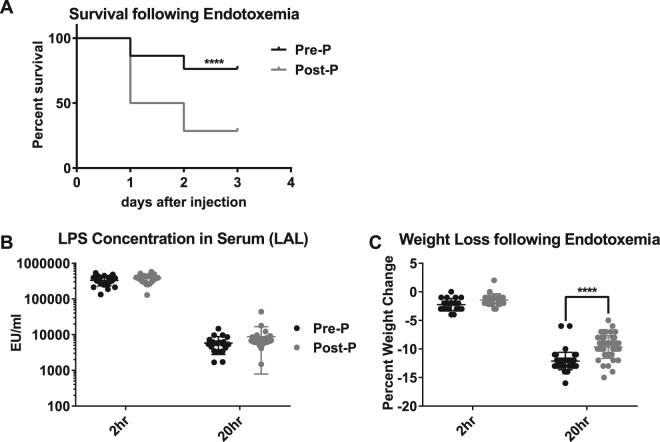
Pre-pubertal mice show increased resistance to mortality from endotoxemia. **(A)** Pre-pubertal (Pre-P) and post-pubertal (Post-P) C57Bl/6 mice received 23 μg/mg LPS via intra-peritoneal injection. Mice were monitored twice daily and moribund individuals humanely euthanised (N ≥ 56/group). **(B)** Endotoxin concentrations in serum samples collected at 2- and 20-hour time-points were quantified using LAL (N ≥ 21/group). **(C)** Mouse weight was recorded prior to LPS injection and then at 2- and 20-hour time points. Data is shown as the per cent change in weight in comparison to time 0 (N ≥ 20/group). Per cent survival was compared using a log rank Mantel Cox test, while endotoxin concentration and weight loss were compared using Two-way ANOVA followed by Tukey’s multiple comparisons test. Significant differences between pre- and post-pubertal mice are labelled with ****(p < 0.0001).

Using an optimised intra-peritoneal LPS injection protocol, we found that female pre-pubertal mice exhibited significantly greater survival than post-pubertal mice over a 72-hour period (6 experiments; N ≥ 56/group; p < 0.0001) (Fig. 1A). Average pre-pubertal survival was 76.3%, while post-pubertal survival was only 28.6%. Serum concentrations of endotoxin at both early (2-hour) and late (20-hour) time points were similar in both pre- and post-pubertal animals (Fig. 1B). This suggests that both age groups were exposed to the same effective endotoxin concentration throughout the experiment. Along with improved survival, pre-pubertal mice also exhibited higher per cent weight loss compared to post-pubertal mice (Fig. 1C). Greater weight loss towards the beginning of the disease course is a common finding associated with survival in mouse models of sepsis^17,18^.

**Figure 2:**
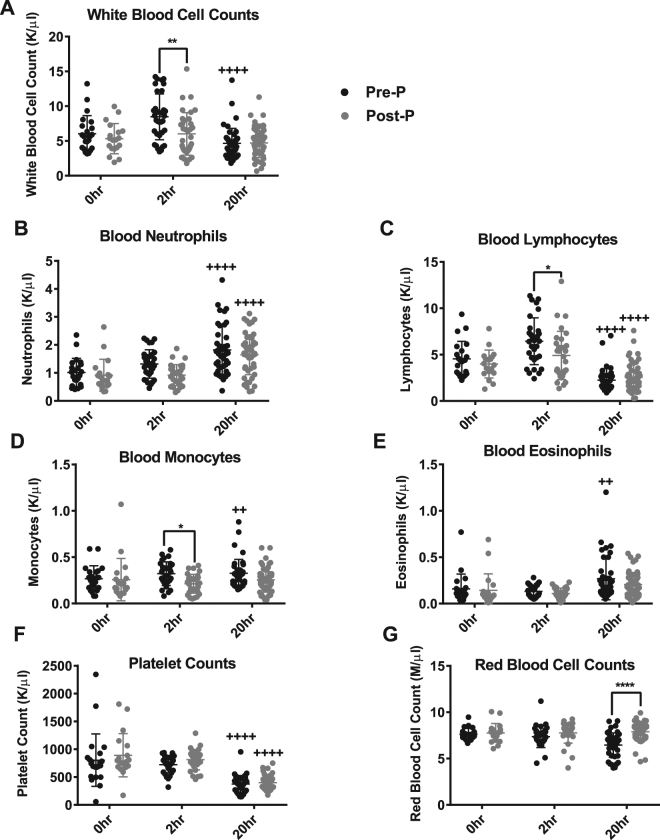
Pre- and post-pubertal whole blood cell counts during endotoxemia. Blood was obtained from pre- and post-pubertal (Pre-P, Post-P) C57Bl/6 mice via cardiac puncture and treated with K_2_EDTA to prevent clotting. All counts for total **(A)** white blood cells, **(B)** neutrophils, **(C)** lymphocytes, **(D)** monocytes, **(E)** eosinophils, **(F)** platelets, and **(G)** red blood cells and were obtained using the Hemavet 950 blood analyser. N ≥ 19/group at 0hr, N ≥ 35/group at 2 hr, and N ≥ 46/group at 20 hr. Significant changes in cell counts between 2 and 20 hours for either pre- or post-pubertal mice are labelled with ^++++^(p < 0.0001) or ^++^(p < 0.01). Significant differences in cell counts between pre- and post-pubertal mice are labelled with ****(p < 0.0001), **(p < 0.01), or *(p < 0.05). All comparisons were made using Two-way ANOVA followed by Tukey’s multiple comparisons test.

To characterise the systemic response to endotoxemia, we assessed the cellular composition of whole blood in pre- and post-pubertal mice. White blood cell (WBC) counts and differentials were similar at baseline in pre- and post-pubertal animals. Two hours after LPS injection, the total WBC count of the pre-pubertal mice spiked above that of the post-pubertal mice (8.5 ± 3.3 K/µl vs. 6.0 ± 3.1 K/µl; p = 0.001) before decreasing at 20 hours to levels matching those of post-pubertal animals (Fig. 2A). The elevated white blood cell count in pre-pubertal animals was associated with increased numbers of lymphocytes and monocytes, but not neutrophils or eosinophils (Fig. 2B–D). At the 20-hour time point, both age groups experienced a significant, but similar, increase in the per cent composition of blood neutrophils, monocytes, and eosinophils and a decrease in the per cent composition of blood lymphocytes (Figure S1a–d). In particular, pre-pubertal animals had higher percentages of monocytes and lower percentages of lymphocytes in comparison to post-pubertal animals. While both age groups experienced thrombocytopenia, only pre-pubertal animals showed a significant decline in RBC count over time (6.5 ± 1.3 M/µl vs. 7.9 ± 1.0 M/µl; p < 0.0001) (Fig. 2F,G). These findings suggest that there are systemic differences in the way pre- and post-pubertal mice react to endotoxin.

Death from sepsis in both humans and mouse models is typically due to injury and failure of important organ systems such as the liver and kidneys^19,20^. To identify any age-associated differences in the accumulation of injury to these organs, we measured serum levels of well-known injury markers: lactate dehydrogenase (LDH), aspartate aminotransferase (AST), and urea. There were some small age-associated differences at the 2-hour time point for serum concentrations of LDH and AST, but not urea. Pre-pubertal animals demonstrated higher serum concentrations of LDH (N = 12/group; 293.2 ± 166.4 U/L vs. 136.3 ± 76.3 U/L; p = 0.01) while post-pubertal animals demonstrated higher serum concentrations of AST (N = 12/group; 66.1 ± 20.4 U/L vs. 37.3 ± 15.8 U/L; p = 0.04). However based on the baseline levels of these injury markers available in the literature, the results for both age groups are well within normal physiological range. This suggests that differences in these values can tell us little about the aetiology of pre-pubertal resistance. Metabolic dysfunction is also an important driving factor in sepsis outcomes^21–24^. To elucidate any possible age-associated differences in metabolic function, we compared the concentrations of glucose and several lipid metabolites (triglyceride, free fatty acids, and cholesterol) in the serum of pre- and post-pubertal animals. We found no age-associated differences (Figure S2a–d).

**Figure 3:**
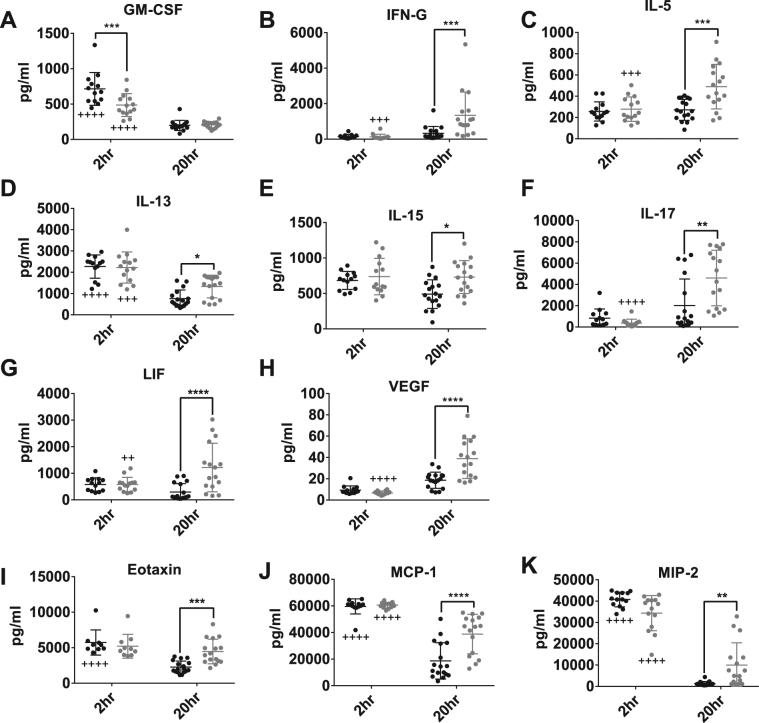
Serum cytokine expression suggests lack of resolution in post-pubertal animals. Serum samples obtained via cardiac puncture from pre- and post-pubertal C57Bl/6 mice (N ≥ 9/group) were subjected to a 32-plex cytokine assay. The 12 cytokines above show significant differences in expression between pre- and post-pubertal mice at either the **(A)** 2-hour time point or **(B–K)** 20-hour time point. For all graphs, pre- and post-pubertal data are shown in black and grey closed circles respectively. Data points above or below the detectable limit of the assay were not included. Significant age-associated differences in cytokine concentration are labelled with ****(p < 0.0001), ***(p < 0.001), **(p < 0.01), or *(p < 0.05). Significant changes in concentration between 2 and 20 hours for either pre- or post-pubertal mice are labelled with ^++++^(p < 0.0001), ^+++^(p < 0.001), or ^++^(p < 0.01). All comparisons were made using Two-way ANOVA followed by Tukey’s multiple comparisons test.

In both animal models and human patients, sepsis severity and mortality risk are tightly associated with elevated expression of both pro- and anti-inflammatory cytokines. To compare cytokine expression in pre- and post-pubertal mice following endotoxemia, serum samples from both age groups at 2 and 20-hour time points were subjected to multiplex cytokine analysis. At the 2-hour time point, pre- and post-pubertal mice showed similar expression of all cytokines except GM-CSF, which was more highly expressed in pre-pubertal mice (714.2 ± 232.8 pg/ml vs. 485.8 ± 161.4 pg/ml; p = 0.0005) (Fig. 3A and Figure S3). At the 20 hr time point, post-pubertal animals exhibited higher expression of many cytokines (IFN-γ, IL-5, IL-13, IL-15, and IL-17) (Fig. 2B–F), growth factors (LIF and VEGF) (Fig. 2G,H), and chemokines (eotaxin, MCP-1, and MIP-2) (Fig. 2I–K) in comparison to their pre-pubertal counterparts. Overall, the temporal trends in cytokine expression were substantially different in pre- and post-pubertal animals. Specifically, while cytokine expression was similar early in endotoxemia, pre-pubertal animals exhibited greater dampening of cytokine expression as time passed.

**Figure 4:**
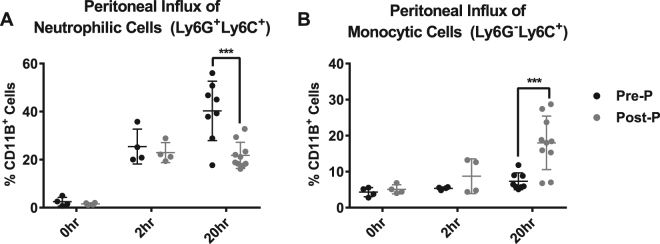
Pre- and post-pubertal mice show differential recruitment of monocytic and neutrophilic cells into the peritoneal cavity. At 2 and 20 hours following endotoxemia, peritoneal cells from pre- and post-pubertal (Pre-P, Post-P) C57Bl/6 mice were collected through peritoneal lavage. Cells were blocked to prevent non-specific binding and treated with antibodies targeting the myeloid lineage marker CD11B, the neutrophilic marker Ly6G, and the monocytic marker Ly6C for subsequent immuno-phenotypic analysis by flow cytometry. (N = 4/group at 0 hr; N = 4/group at 2 hr; N = 8/group at 20 hr). Pre- and post-pubertal mice exhibited different temporal patterns in the influx of **(A)** neutrophilic and **(B)** monocytic cells. For each sample, a total of ten thousand cells were analysed using the gating strategy described in Figure S4. Significant differences in concentration between pre- and post-pubertal mice are labelled with ***(p < 0.001). All comparisons were made using Two-way ANOVA followed by Tukey’s multiple comparisons test.

As assessed by flow cytometry, in both age groups, endotoxin injection caused an influx of leukocytes into the peritoneal cavity. Among CD11B^+^ cells (myeloid lineage), pre-pubertal mice exhibited a higher percentage of neutrophilic Ly6G^+^Ly6C^+^ cells and a lower percentage of monocytic Ly6G^−^Ly6C^+^ cells, when compared to post-pubertal mice. These differences were significant at the 20-hour time point (Fig. 4A,B). Between 0 and 2 hours, there were substantial increases in neutrophilic cells within the peritoneal cavities of both pre- and post-pubertal animals (~20%). However, while the per cent composition continued to rise from 2 to 20 hours in pre-pubertal animals (25.5% ± 7.3% vs. 40.3% ± 12.4%; p = 0.03), it did not change in post-pubertal animals (22.9% ± 4.1% vs. 21.8% ± 5.4%) (Fig. 4A). While there were no significant changes in the peritoneal influx of monocytic cells for either age between 0 and 2 hours, post-pubertal animals exhibited a significant increase in this cell type from 2 to 20 hours (8.7% ± 4.8% vs. 18.0% ± 7.4%; p = 0.025), while pre-pubertal animals did not (5.4% ± 0.4% vs. 7.3% ± 2.3%) (Fig. 4B). Though there were other changes in peritoneal cell composition in response to endotoxin, none demonstrated an age-associated trend (Figure S5). The gating strategy used for these experiments (Figure S4) did not include a live-dead exclusion, only a removal of doublet cells. We had previously evaluated viability in a limited number of samples and determined that, the percentage of dead (7AAD^+^) cells was equal in both age groups at both the 2 (~15%) and 20-hour (~45%) time points. The differential recruitment of neutrophils and monocytes to the peritoneal cavity suggests an underlying age-associated difference in the regulation of endotoxin-induced inflammation.

### Hormonal manipulation of pubertal status alters survival from endotoxemia

**Figure 5:**
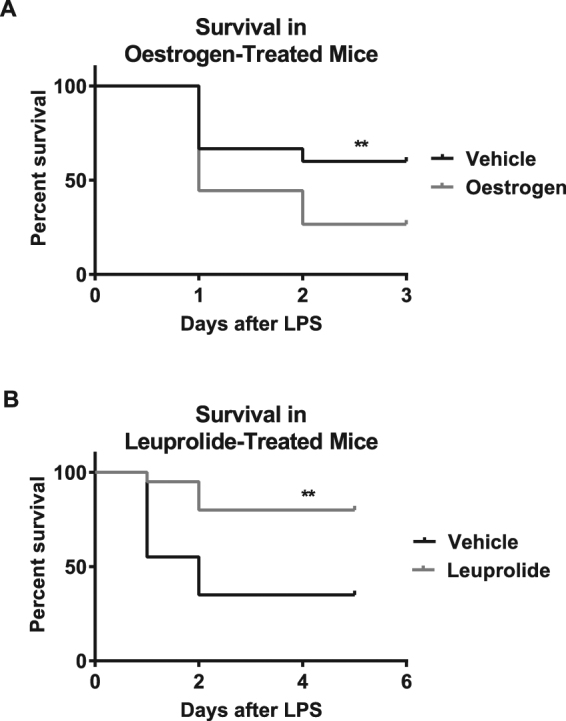
Delay or expedition of puberty by hormonal treatment alters mortality from endotoxemia. (**A)** Pre-pubertal CD-1 mice were pre-treated with daily subcutaneous injections of 17β-Oestradiol at 100 μg/ml or vehicle (0.4% DMSO) suspended in corn oil for three days prior and once on the day of intraperitoneal *E. coli* endotoxin injection. Per cent survival over three days was recorded (N = 45/group). **(B)** Pre-pubertal C57Bl/6 mice were treated with daily subcutaneous injections of leuprolide at 250 μg/ml or vehicle (0.9% sodium chloride) starting on PND 24 and continuing until post-puberty (PND 35) before *S. enterica* endotoxin injection (N = 20/group). Per cent survival over three days was recorded. Significant differences in survival between pre- and post-pubertal mice are labelled **(p < 0.01). Per cent survival was compared using a log rank Mantel Cox test.

We next investigated how sex hormones and pubertal status influence the differential mortality of pre- and post-pubertal animals in response to endotoxemia. Specifically, we tested the postulate that the increased oestrogen production that accompanies puberty onset promotes the susceptibility of post-pubertal animals. Pre-treatment of pre-pubertal mice with oestrogen prior to endotoxemia significantly expedited vaginal opening (an early indicator of female mouse puberty; Figure S6) and increased mortality from endotoxemia compared to vehicle-treated mice (4 experiments; N = 45/group; 60% vs. 26.7%; p = 0.002) (Fig. 5A). In a complementary strategy, we prevented the onset of puberty by pre-treatment with the gonadotropin releasing hormone (GnRH) agonist leuprolide, which desensitises the GnRH receptor and prevents the release of pubertal gonadotropins, (e.g. luteinising hormone). This intervention led to improved survival compared to age-matched controls (3 experiments; N = 20/group; 80% vs. 35%; p < 0.0001) (Fig. 5B). To determine whether increased mortality was specifically related to the onset of puberty or to a lack of oestrogen activity in general, prior to endotoxemia we also pre-treated post-pubertal mice with the oestrogen-receptor agonist fulvestrant. Fulvestrant did not reliably improve survival compared to vehicle-treated controls (Figure S7). These findings suggest that oestrogen is important in driving pre-pubertal resistance to endotoxemia mortality, especially during initiation of puberty (see Discussion).

### Adoptive transfer of pre-pubertal peritoneal cells improves survival from endotoxemia

**Figure 6:**
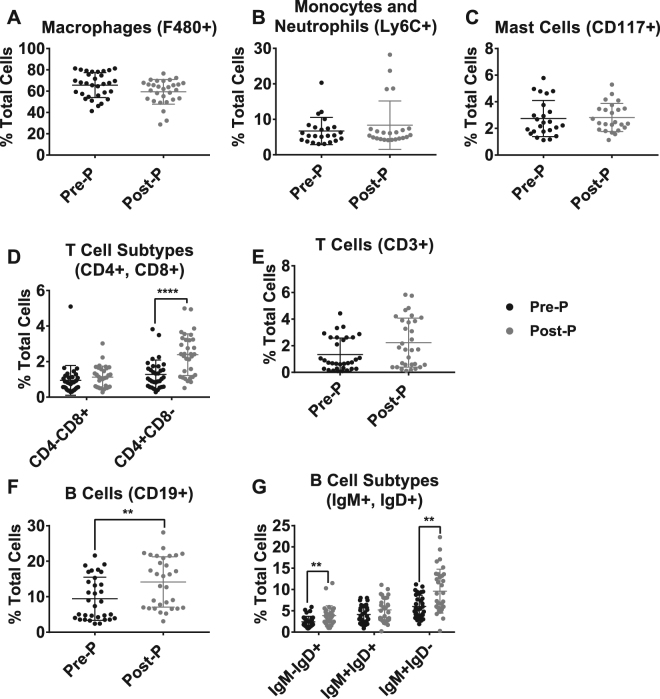
Pre- and post-pubertal (Pre-P, Post-P) mice show similar naïve peritoneal cell profiles. Naïve peritoneal cells from pre- and post-pubertal C57Bl/6 mice were collected through peritoneal lavage. Cells were blocked to prevent non-specific binding and then treated with antibodies for subsequent immuno-phenotypic analysis by flow cytometry. These included **(A)** F4/80^+^ Macrophages, **(B)** Ly6C^+^ Monocytes and Neutrophils, **(C)** CD117^+^ Mast Cells, **(D)** CD3^+^ T cells and associated **(E)** CD4^+^ and CD8^+^ subsets and **(F)** CD19^+^ B cells and associated **(G)** IgM^+^ and IgD^+^ subsets. Black closed circles signify pre-pubertal data. N ≥ 23/group. For each sample, a total of ten thousand cells were analysed using the gating strategy described in Figure S8. Significant differences in concentration between pre- and post-pubertal mice are labelled with **(p < 0.01). All comparisons were made using the Mann Whitney test.

Before treatment with endotoxin, pre- and post-pubertal mice exhibited similar peritoneal cell profiles, as assessed by flow cytometry (Fig. 6A–G) (Figure S8). Though there was a slightly higher per cent composition of F4/80^+^ macrophages in pre-pubertal mice (Fig. 6A) and CD3^+^ T cells in post-pubertal mice (Fig. 6D), neither of these differences was significant. However, there was a significantly higher per cent composition of CD19^+^ B cells of both IgD^+^ and IgM^+^ subsets in the peritoneal cavity of post-pubertal mice (Fig. 6F,G). In addition, the per cent composition of the CD4^+^ subset of T cells was higher in post-pubertal animals.

**Figure 7:**
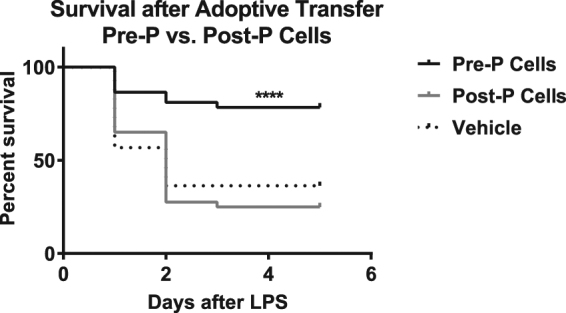
Adoptive transfer of pre-pubertal cells improves post-pubertal survival. Naïve peritoneal cells were collected from pre-pubertal mice or post-pubertal mice by peritoneal lavage. Recipient post-pubertal mice were administered 1 mL of pre-pubertal (Pre-P) or post-pubertal (Post-P) peritoneal cell suspensions (2 million cells total), or 1 mL of the vehicle (PBS) via intraperitoneal injection. Following incubation of donor cells within the peritoneal cavity, the recipient mice were then subjected to endotoxemia with either *S. enterica* or *E. coli* endotoxin (N ≥ 37/group). Per cent survival was recorded over five days. Significant differences in survival between mice administered Pre-P cells vs. those receiving Post-P cells or Vehicle were ****(p < 0.0001). Per cent survival was compared using a log rank Mantel Cox test.

We wanted to determine whether adoptive transfer of resident pre-pubertal cells could improve the resistance of post-pubertal animals to endotoxemia-induced mortality. Adoptive transfer of resident peritoneal cells from pre-pubertal mice into recipient post-pubertal mice, markedly increased survival after endotoxemia, while transfer of post-pubertal cells or vehicle did not (4 experiments; N ≥ 37/group; 78.3% vs. 25.0% vs. 36.4%; p < 0.0001) (Fig. 7). These findings indicate that the “pre-pubertal resistance” can be transferred to a post-pubertal animal and that peritoneal immune cells are intimately involved in this transference.

We sought to determine which cell subpopulation(s) contributed to the adoptive transfer findings. We used a magnetic bead-based system (Miltenyi MACS) to perform negative selection of different cell types from the pre-pubertal peritoneal cells and then used the separated cells for adoptive transfer. Adoptive transfer with negatively-selected macrophages (F4/80^+^) or negatively-selected combined B and T cells (CD19^+^; CD3^+^), was unable to confer the same survival benefit as treatment with the non-separated peritoneal cell mix. However, even when separated cells were recombined prior to adoptive transfer no survival benefit was gained (Figure S9). These findings suggest that the separation method interfered with the functionality of the cells.

## Discussion

Epidemiological data indicate that human children, particularly in the pre-pubertal age-range, exhibit a striking but understudied resistance to mortality from sepsis. In this study, we used a mouse model of endotoxemia to characterise the phenomenon of childhood resistance to sepsis mortality and begin to explore its underlying mechanisms. Pre-pubertal mice showed a highly significant resistance to mortality from endotoxemia in comparison to post-pubertal mice, despite having equally robust initial responses in cytokine production and leukocyte dynamics. Age-associated differences in the response to endotoxemia were most evident at the later 20-hour time point, when post-pubertal mice showed continued elevation of cytokines and monocytic, rather than neutrophilic, influx of cells into the peritoneal cavity. Blocking puberty using leuprolide or acceleration of puberty using oestrogen led to increased or decreased survival from endotoxemia, respectively. In addition, the adoptive transfer of pre-pubertal, but not post-pubertal, peritoneal cells improved the survival of post-pubertal recipient mice, suggesting that specific pre-pubertal cell type(s), rather than pre-formed mediators or cells elsewhere (e.g. endothelium) might be functionally sufficient to mediate protection from mortality.

The endotoxin injection or “endotoxemia” model of sepsis has important limitations that merit discussion. This model focuses entirely on the role that the host response plays in driving the pathophysiology of sepsis. It brings about rapid onset of systemic clinical signs (e.g. reduced motor activity, pilo-erection, hypothermia, etc.)^20,25,26^; a hypo-dynamic cardiac state characterised by decreased cardiac output and decreased peripheral vascular resistance; decreased total white blood cell counts (with marked reduction in lymphocytes and neutrophils); and a rapid, but transient increase in systemic cytokine levels^26,27^. In our studies, some variability from trial to trial was observed in the absolute level of mortality achieved, which may reflect some of the variables known to affect mouse models (e.g. time, location, handling in vivarium, seasonal effects, etc.). Nevertheless, the age-associated differences seen in this study were robust to these variations and were consistently observed.

While some of the manifestations of acute endotoxemia in mice mirror those in human sepsis, such as the simultaneous release of pro- and anti-inflammatory mediators^28–31^, the clinical course and progression of disease in rodent endotoxemia models is much faster in mice than in humans with polymicrobial sepsis or low-grade endotoxemia^27^. Additionally, mice are relatively insensitive to LPS and require high doses to produce a shock-like state (nearly 250 times that in humans^25^). More broadly, there are well recognised problems in the numerous models attempting to replicate the complexities of sepsis (e.g. cecal ligation and puncture (CLP)), each with its own advantages and limitations^20,27,32^. The mice typically used in sepsis studies are inbred, male, young, healthy, and given minimal to no supportive care (e.g. antibiotics, vasopressors, fluid resuscitation, ventilation etc.). It is perhaps no surprise that these animal subjects would differ so strongly in their physiology from human sepsis patients who are of both sexes, over the age of 65, often suffering from co-morbidities, and given aggressive supportive care^33,34^. Beyond issues with creating more representative models of sepsis in mice, there are fundamentally significant species differences between mice and humans. There have been multiple attempts to compare the transcriptional regulation in mice and humans, but the results are contentious^35^. For example, a publication by Seok *et al*. (2013) shook the sepsis field when it found no correlation between the transcriptional networks of human and murine leukocytes following burns, trauma, and endotoxin exposure^36^, findings that were later contextualised in a more positive light by Takao *et al*.^37^.

Extrapolations from the endotoxemia model can often favour the identification of treatments that dampen exuberant inflammation but ultimately do not improve survival in more complex human sepsis^2^. However, in our study the improved survival of pre-pubertal mice did not appear to be a result of an inadequate or dampened initial response to immune stimulation by endotoxin. At two hours, pre- and post-pubertal mice exhibited equally robust inflammatory responses to endotoxin in both serum cytokine production and leukocyte recruitment. This finding, along with the detection of equal endotoxin concentrations in the sera of both age groups, suggest that the survival benefit of pre-puberty is not based on an initially weaker host response. It is worth noting that saline was given prophylactically at the time of endotoxin administration but was not provided in the later stages of illness. Though the merits of fluid resuscitation in mouse models of sepsis are well established^38,39^ we wanted to avoid the stress that subcutaneous injections would have on the sick animals. The illness induced by endotoxemia is so rapid that continued handling of animals can alter survival. Considering that pre-pubertal survival from endotoxemia was associated with robust initial responses to endotoxin, we hypothesise that pre-pubertal animals will also survive better in live bacterial models of sepsis (e.g. CLP, fibrin clot peritonitis, etc.). Given the increasing recognition of the limitations of murine models of sepsis, future studies might benefit by using a larger animal model, the rabbit, which has a sensitivity to low doses of endotoxin similar to that seen in people. We note that a similar resistance to mortality from LPS in pre-pubertal vs. pubertal rabbits has been reported^40^, although this finding was not the focus of the study.

Twenty hours after endotoxin administration, key differences between pre- and of post-pubertal mice became apparent in both the regulation of cytokine expression and peritoneal leukocyte composition. Post-pubertal animals exhibited sustained serum levels of a number of cytokines, chemokines and growth factors commonly associated with poor outcomes in sepsis (Fig. 3). Surprisingly most of the canonical sepsis cytokines^41^ (e.g. interleukin 10 (IL-10), IL-1α, IL-1β, tumour necrosis factor α (TNF-α)) were expressed equally in both age groups (Figure S3). Interestingly, the majority of differentially regulated cytokines were associated with T cell biology. These cytokines included Th1 (interferon γ (IFN-γ)), Th2 (IL-5 & IL-13) and Th17 (IL-17) type cytokines, as well as IL-15, a T cell growth factor expressed by mononuclear cells. Post-pubertal mice also exhibited increased expression of three chemokines: eotaxin 1 (Eotaxin), monocyte chemotactic protein 1 (MCP-1), and macrophage inflammatory protein 2 (MIP-2). These three chemokines target the recruitment of eosinophils, inflammatory monocytes and neutrophils respectively. However, there was no clear connection between the differential expression of these chemokines and the composition of leukocytes found in the blood and peritoneal cavity. Finally, the growth factors leukaemia inhibitory factor (LIF) and vascular endothelial growth factor (VEGF) also showed increased expression in post-pubertal serum at 20 hours. LIF is a cytokine that is commonly identified in the serum of critically ill humans and animal models and has both documented pro- and anti-inflammatory functions^42–45^. Among other functions, VEGF is extremely important in the regulation of vascular permeability. Overexpression of this factor, paired with the finding of diminished weight loss in post-pubertal animals (Fig. 1C), suggest that vascular leak and oedema may have contributed to poor outcomes in this group^18,20,46^. We note that our experimental design may have precluded the detection of important differences in inflammatory parameters between the time points of 2 and 20 hours. For example, many cytokines continue to fluctuate between 2 and 8 hours after endotoxemia^25,26^. By increasing the density of time points in future studies, we might better capture the events driving resolution in pre- vs. post-pubertal animals.

In addition to cytokine expression, there were substantial changes to the cellular composition of the peritoneal cavity. While both pre- and post-pubertal mice had an influx of neutrophilic CD11B^+^ cells between 0 and 2 hours following endotoxemia, only pre-pubertal animals showed continued recruitment through to 20 hours. In contrast, only post-pubertal animals showed a marked increased in the per cent composition of monocytic CD11B^+^ cells between 2 and 20 hours. The basis for this preferential recruitment of monocytic cells into the peritoneal cavity is unclear, considering that post-pubertal animals expressed high levels of both neutrophilic and monocytic chemokines. It is possible that the incongruence between the elevation of neutrophilic chemokines and the decreased numbers of peritoneal neutrophils in post-pubertal animals is due to impaired neutrophil chemotaxis. Neutrophil dysfunction, a common feature of sepsis in both human patients and mouse models, typically results in the troublesome adhesion and sequestration of activated neutrophils in the intravascular spaces and peripheral, unaffected tissues (e.g. lung, liver, kidney etc.). The mediators released by these activated neutrophils contribute to harmful systemic inflammation, endothelial dysfunction, hypotension, and coagulation^47,48^. An increased presence of activated neutrophils in non-peritoneal tissues may have contributed to the increased mortality seen among post-pubertal animals and is worthy of further investigation, as are time points between the 2 and 20 hours.

This work indicates that oestrogen, and its elevation during puberty, plays a role in driving the differences between pre- and post-pubertal responses to endotoxemia. Hormonal acceleration of puberty through oestrogen pre-treatment increased mortality while the inhibition of puberty with leuprolide decreased mortality. The importance of oestrogen and the pubertal transition is also evident in another model of pre-pubertal resistance explored by our lab using H1N1 influenza infection. Mice subjected to ovariectomy before the onset of puberty had increased survival from infection—a finding that was reversed with oestrogen replacement. In addition, delaying puberty by the administration of leuprolide improved survival of female mice following infection^49^. Despite the apparent importance of oestrogen in driving the loss of resistance after puberty, pre-treatment of post-pubertal mice with the oestrogen-receptor antagonist fulvestrant before endotoxemia did not reliably improve their survival compared to vehicle-treated controls (Figure S7). This contrasts with beneficial effects of fulvestrant seen in the influenza model^49^. One possible explanation for this discordance is that exposure to elevated oestrogen in these animals at ~27–35 days of age before fulvestrant was started was sufficient to cause an irreversible change in susceptibility. Additional studies of the role of pubertal oestrogen in priming animals for poor outcomes during endotoxemia may provide insight. This includes determining the receptor types and target cells involved, as well as directly characterizing the ways in which oestrogen-primed pre-pubertal animals respond similarly to endotoxin as post-pubertal animals.

As evidenced in the endotoxemia data, oestrogen appears to play an important role in disrupting the pre-pubertal resistance to mortality. Although our hormonal manipulation studies used only female mice, the human epidemiology and other experimental data in endotoxemia^40^ and influenza^49,50^ suggest that pre-pubertal resistance to mortality occurs in both sexes. This is logical, considering the essential role that oestrogen plays in the pubertal development of both sexes. Plasma oestrogen levels are extremely low during pre-puberty in both males and females, but begin to rise at the beginning of puberty. Though the rise occurs earlier in girls than in boys, the bioactive oestrogen levels for growth in both sexes are equivalent when growth velocity peaks^51^. The fact that oestrogen has such profound effects on the pubertal development of both sexes suggests that it may also be able to unilaterally alter the susceptibility to death from sepsis. However, future studies (e.g. in rabbit models of sepsis) should include evaluation of the pre-pubertal resistance phenomenon in both sexes.

In our mouse model of endotoxemia, we found that treatment of pre-pubertal animals with oestrogen increased their mortality. In contrast with these findings, there is a sizable body of work in adult rodents that demonstrates the survival benefits of oestrogen in sepsis and other forms of critical illness. Oestrogen (both exogenous and endogenous) has been shown to protect vital organs and improve survival during CLP^52^, a combination of haemorrhage and CLP^53,54^, as well as other rodent models of critical illness^55,56^. However, in the clinical setting, there are conflicting reports as to whether female sex is protective during sepsis^57^. An additional complication in understanding the role of oestrogen in sepsis is the finding that critically ill individuals often have increased serum levels of oestrogen. This is caused by increased rates of peripheral aromatization in response to stress^58^ and is associated with increased risk of mortality^59,60^. The effect of oestrogen on outcomes in sepsis and other critical illnesses is complicated further still by its dynamic, non-monotonic response curve^61^. For example, in the context of immunity, oestrogen can be anti-inflammatory at high concentrations and pro-inflammatory at lower concentrations^62,63^.

In addition to the contribution of oestrogen to the control of pre-pubertal resistance, we established other clues toward possible downstream mechanisms. Specifically, pre-pubertal resistance can be conferred to post-pubertal animals by adoptive transfer of pre-pubertal resident peritoneal cells. The particular biological activity of these cells in driving pre-pubertal resistance remains unknown. We consider the two most numerous cell types as possible mediators: B-cells and macrophages. We favour the former, based in part on the finding that post-pubertal mice have a slightly higher proportion of these cells. Though efforts to test individual cell types purified by magnetic bead separation failed, we determined that the separation methodology itself abolished the protective function of pre-pubertal cells, precluding useful interpretation. Alternative methods (e.g., flow cytometric sorting, use of mice missing specific lineages such as macrophages^64,65^ or B cells^66^ (Ighm^tm1Cgn^, Jackson Labs)) may allow identification of the responsible cell type(s) in future studies.

We can only speculate on how oestrogen might drive the differences between pre- and post-pubertal peritoneal cell populations. There were no notable differences in naive cell concentrations between the pre- and post-pubertal animals, but it is possible that more detailed phenotyping efforts will indicate otherwise. Unanswered questions include whether the population of peritoneal cells changes in oestrogen-treated mice, and which cell types persist in the peritoneal cavity after adoptive transfer. If the pre-pubertal resistance is disturbed directly by the first encounter with higher levels of oestrogen, and occurs equally in males and females, we can postulate that the effect of interest is most likely an action of the hormone occurring in both sexes. The only study available looked at the effects of puberty on splenic transcriptomes in male and female mice and did not note many similarities in the changing patterns of gene expression^67^. Additional data exploring the non-sex-specific effects of puberty on the immune system could help illuminate the mechanisms associated with pre-pubertal resistance.

Our findings suggest that a change occurring at puberty, mediated by increased oestrogen, causes a permanent adjustment in the regulation of the immune system, which results in greater susceptibility to death from endotoxemia. Although aging-associated changes in basal inflammation and oxidative stress may account for some of these differences, our findings suggest that the oestrogen released with the onset of puberty is critical. The data establish a model for childhood resistance to mortality from endotoxin, and identify peritoneal cells as mediators of pre-pubertal resistance that merit additional characterisation.

## Materials and Methods

### Animals

All C57Bl/6 and CD1 mice were obtained from Charles River (Wilmington, MA), housed in micro-isolator Full Sterile Technique (FST) cages, and fed standard chow ad libitum. After delivery, mice were allowed at minimum, a three-day acclimatisation period prior to usage in experiments. Pre-pubertal mice typically arrived in the animal facility on post-natal day (PND) 21 and were used for experiments on PND 24–26. Post-pubertal mice typically arrived in the animal facility on PND 30 and were used for experiments on PND 33–35. Mice were cared for according to the Guide for the Care and Use of Laboratory Animals (NIH) and all animal protocols were approved by the Harvard Centre for Comparative Medicine (HCCM).

### Endotoxemia Model of Sterile Sepsis

Female pre- and post-pubertal mice were given intra-peritoneal injections of *E.coli* LPS (L3755, Sigma; Lots: 123M4096V; 066M4118V) diluted in sterile water followed by a prophylactic fluid resuscitation of 0.9% sodium chloride delivered subcutaneously in the scruff of the neck, equal to 2.5% of the animal’s body weight. No other fluid resuscitation was performed to minimise stress upon the sick animals. The concentrations of LPS used to elicit acute endotoxemia differed depending on the potency of the particular lot of product. Lot 123M4096V achieved effective mortality in a range of 23–25 μg/g while Lot 066M4118V did so at a dosage of 30–32 μg/g. Dose response experiments were performed whenever a new lot of LPS was used to determine the 80–90% lethal dose. To avoid diurnal differences in metabolism and immune function^68–70^, all LPS injections were administered between 11:00 and 14:00. After injections, mice were monitored twice daily for weight-loss, mortality and signs of significant morbidity including stressed posture, lack of movement and laboured breathing. Mice determined to be moribund were euthanised via intra-peritoneal injection with a 1:4 dilution of Fatal Plus (pentobarbital sodium solution) (Vortech; NDC0298–9373–68) in PBS. Typically, experiments ended after 72 hours. For each time course study, in addition to the mouse subjects sacrificed for sample collection at 0, 2 and 20 hours, N ≥ 5 mice from each age group were set aside in order to evaluate survival and determine whether the “pre-pubertal resistance” criterion was being met in each experiment.

In studies involving oestrogen pre-treatment, pre-pubertal mice were treated with daily subcutaneous 100ul injections of 17β-Oestradiol at 100 μg/ml^71^ (Cayman; 10006315) or vehicle (0.4% DMSO) suspended in corn oil for three days prior and once on the day of endotoxin injection. In studies involving fulvestrant pre-treatment, post-pubertal mice were treated with daily subcutaneous 100ul injections of fulvestrant (Sigma; I4409) at 2 mg/ml or vehicle (0.8% DMSO) in corn oil for three days prior and once on the day of endotoxin injection. CD-1 mice were used in the oestrogen and fulvestrant pre-treatment studies. In studies involving leuprolide pre-treatment, pre-pubertal mice were treated with daily subcutaneous 100ul injections of leuprolide (Tocris Bioscience-2873) at 250 μg/ml or vehicle (0.9% sodium chloride) starting on PND 24 and continuing until PND 35 before endotoxin injection. For this particular experiment, which was originally part of an early pilot series, *Salmonella enterica* LPS (Sigma; L2262; Lot: 081M4034) was used at 32 μg/g.

### Blood and Serum Analyses of Endotoxemic Mice

During endotoxemia time course studies, mice were euthanised 0, 2 or 20 hours after LPS injection by inhaled isoflurane overdose. Blood was collected via cardiac puncture and transferred into lavender-top K_2_EDTA tubes and gold-top serum separation tubes and inverted to mix. Anti-coagulated EDTA-treated blood was used for red and white blood cell counts and differentials assessed using the Hemavet 950 (Drew Scientific). Gold top tubes were centrifuged for 10 min and the isolated serum frozen at −20 °C. Serum cytokines were quantified via multiplex analysis (Mouse Cytokine Array/Chemokine array 32-plex) performed by EVE Technologies (Calgary, Alberta, Canada). Serum endotoxin concentrations were quantified using two different methods: 1) Pierce LAL Chromogenic Endotoxin Quantitation Kit (ThermoFisher Scientific-88282) and 2) PyrogentTM 5000 Kinetic Turbidimetric LAL Assay (Lonza; N383). Serum levels of injury markers aspartate aminotransferase (AST), lactate dehydrogenase (LDH), and urea were quantified using commercial kits. These included InfinityTM AST Liquid Stable Reagent (Thermo Scientific), Lactate Dehyrdrogenase (Liquid) Reagent Set (Pointe Scientific, Inc.), and Liquid Urea Nitrogen (BUN) Reagent Set (Pointe Scientific Inc.). Serum analysis of metabolic analytes including free fatty acids (FFA), triglycerides (TG), and cholesterol (CL) were performed using commercial kits. These include Wako HR series NEFA-HR (Wako Diagnostics), InfinityTM Cholesterol Liquid Stable Reagent (Thermo Scientific), and InfinityTM Triglycerides Liquid Stable Reagent (Thermo Scientific). Serum glucose was assessed using the Easy Step Blood Glucose Monitoring System (Easy Step). To measure blood glucose, mice were securely held and a razor blade used to elicit a small drop of blood from the tip of the animal’s tail. This drop of blood was put into contact with the test strip, allowing the blood to be carried into the analyser by capillary action and the glucose concentration calculated. Blood flow from animal’s tail was stopped by gentle application of pressure with gauze and styptic powder.

### Peritoneal Lavage

At 0, 2, or 20 hours following LPS injection, mice were euthanised and subjected to peritoneal lavage. Briefly, surgical scissors and forceps were used to make an incision in the skin and reveal the peritoneal wall. A syringe outfitted with a 27 G needle was used to inject 5 mL of PBS (4 °C) into the peritoneal cavity. The fluid-filled peritoneal cavity of each mouse was gently mixed by massaging for one minute. The lavage fluid was then drawn out of the peritoneal cavity using a syringe outfitted with a 22 G needle. Lavage fluid was centrifuged at 300 G for 10 minutes and resuspended in the appropriate assay buffer.

### Immuno-phenotyping of Peritoneal Cells

For immuno-phenotyping analyses by flow cytometry, isolated peritoneal cells were resuspended in cell staining buffer (Biolegend; 420201), incubated for ten minutes at 4 °C with Tru-Stain fcX^TM^ (anti-mouse CD16/32) antibody (Biolegend; 101320) to prevent non-specific binding, and finally incubated with multiple combinations of fluorescent antibodies for 15 minutes at 4 °C (Table S1). Cells were collected on a BD FACSCantoII^TM^ using the BD FACSDIVA Software. For each sample, a total of ten thousand cells were analysed. Data were further analysed using Flow-Jo®.

### Adoptive Transfer Studies

Naïve peritoneal cells were collected from pre-pubertal mice or post-pubertal mice by peritoneal lavage as described above. Lavage fluid was centrifuged at 300 G for 10 minutes and resuspended in PBS (4 °C) at a concentration of 2 million total cells/ml. Recipient post-pubertal mice were administered either 1 mL of pre- or post-pubertal peritoneal cell suspensions (2 million cells total) or 1 mL of PBS (i.p.). Following 30–60 minutes of incubation within the peritoneal cavity, the recipient mice were then subjected to the endotoxemia protocol described above. Three of the four initial adoptive transfer experiments were performed using *Salmonella enterica* LPS instead of *E. coli* LPS. The results (pooled in Fig. 7) did not differ based on the source of endotoxin used.

### Adoptive Transfer of Magnetically Separated Peritoneal Cells

Naïve peritoneal cells were collected from pre-pubertal mice. Specific cell types were separated from the pre-pubertal peritoneal cell suspensions prior to adoptive transfer using the Miltenyi MACS® system. Briefly, following lavage and centrifugation, peritoneal cells were resuspended in MACS® buffer (PBS pH 7.2; 0.5% BSA, 2 mM EDTA) at 1 million cells/ml and incubated (15 minutes; 4 °C) with either biotinylated F4/80 antibody (Biolegend; 123106) or a cocktail of biotinylated CD3 and CD19 antibodies (Biolegend; 100243; Biolegend; 115503). This methodology allowed for positive or negative selection of macrophages and T cells/ B cells respectively. After incubation, cells were washed twice and then resuspended in 80 μl MACS Buffer. 20 μl of anti-biotin microbeads (Miltenyi; 130-090-485) were added to the cell suspension and incubated for 15 minutes on ice. Cells were washed once and resuspended in 500 μl MACS buffer before application to the LS Column (Miltenyi; 120-042-401) within the magnetic field of the QuadroMACS Separator. The column was washed three times with MACS buffer and the untagged cells collected in the flow through. The column was then removed from the magnet and the tagged cells were washed from the column. Separated cells and flow-through cells were each centrifuged at 300 G for 10 minutes and resuspended in PBS for adoptive transfer. Separated macrophages were administered at 1.5 million cells/ml, the T and B cell mix at 0.5 million cells/ml, recombined cell mix at 2 million cells /ml, or un-separated cell mix at 2 million cells/ml.

### Statistical Analyses

All statistical analyses were performed using Prism 7 (Graphpad). The methodologies used and statistical significance are detailed in the figure legends. Data is presented in the results section as the mean ± the standard deviation.

## Electronic supplementary material

Supplementary Information
