# Supplementary material for: Characterising Pre-pubertal Resistance to Death from Endotoxemia
Source: Sci Rep. 2017 Nov 29;7:16541. doi: 10.1038/s41598-017-16743-1 (PMC5707402; doi:10.1038/s41598-017-16743-1)
Supplement: Supplementary file 1 — Supplementary Information [file 41598_2017_16743_MOESM1_ESM.pdf]

Supplementary Information for:

Characterising Pre-pubertal Resistance to Death from Endotoxemia

Rose Joachim[1], Freeman Suber[1], Lester Kobzik[1]*

[1]Harvard T. H. Chan School of Public Health, Boston, MA, USA

*Corresponding Author: lkobzik@hsph.harvard.edu

Supplementary Materials and Methods

Table S1. Antibodies used for flow cytometry experiments.

Figure S1. Pre and post-pubertal white blood cell composition during endotoxemia

Figure S2. Serum metabolic factors are similar in endotoxemic pre- and post-pubertal mice

Figure S3. Serum cytokine expression following endotoxemia

Figure S4. Flow cytometry experiment gating strategy and representative dot plots used for assessing endotoxin-induced changes to peritoneal cell composition.

Figure S5. Changing peritoneal cell profiles in response to endotoxemia

Figure S6. Vaginal opening following treatment with oestrogen or vehicle

Figure S7. Effects of fulvestrant pre-treatment on endotoxemia survival

Figure S8. Flow cytometry experiment gating strategy and representative dot plots used for naïve peritoneal cell phenotyping.

Figure S9. Adoptive transfer of pre-pubertal peritoneal cells separated using magnetic beads failed to improve survival

Specificity	Clone Identifier	Fluorochrome	Vendor	Product Number
CD11B	M1/70	Alexafluor 647	Biolegend	101218
CD11B	M1/70	APC	Biolegend	101212
CD19	6D5	APC	Biolegend	115512
CD3	17A2	APC	Biolegend	100236
F4/80	BM8	FITC	Biolegend	123107
Ly6C	HK1.4	FITC	Biolegend	128005
IgM	RMM-1	FITC	Biolegend	406505
CD8a	53-6.7	FITC	Biolegend	100706
CD117	2B8	PE	Biolegend	105807
Ly6G	1A8	PE	Biolegend	127607
IgD	11-26c.2a	PE	Biolegend	405705
CD4	GK1.5	PE	Biolegend	100408

Table S1. Antibodies used for flow cytometry experiments. The table above contains detailed information regarding the antibodies used for immune-phenotypic analysis of mouse peritoneal cells.

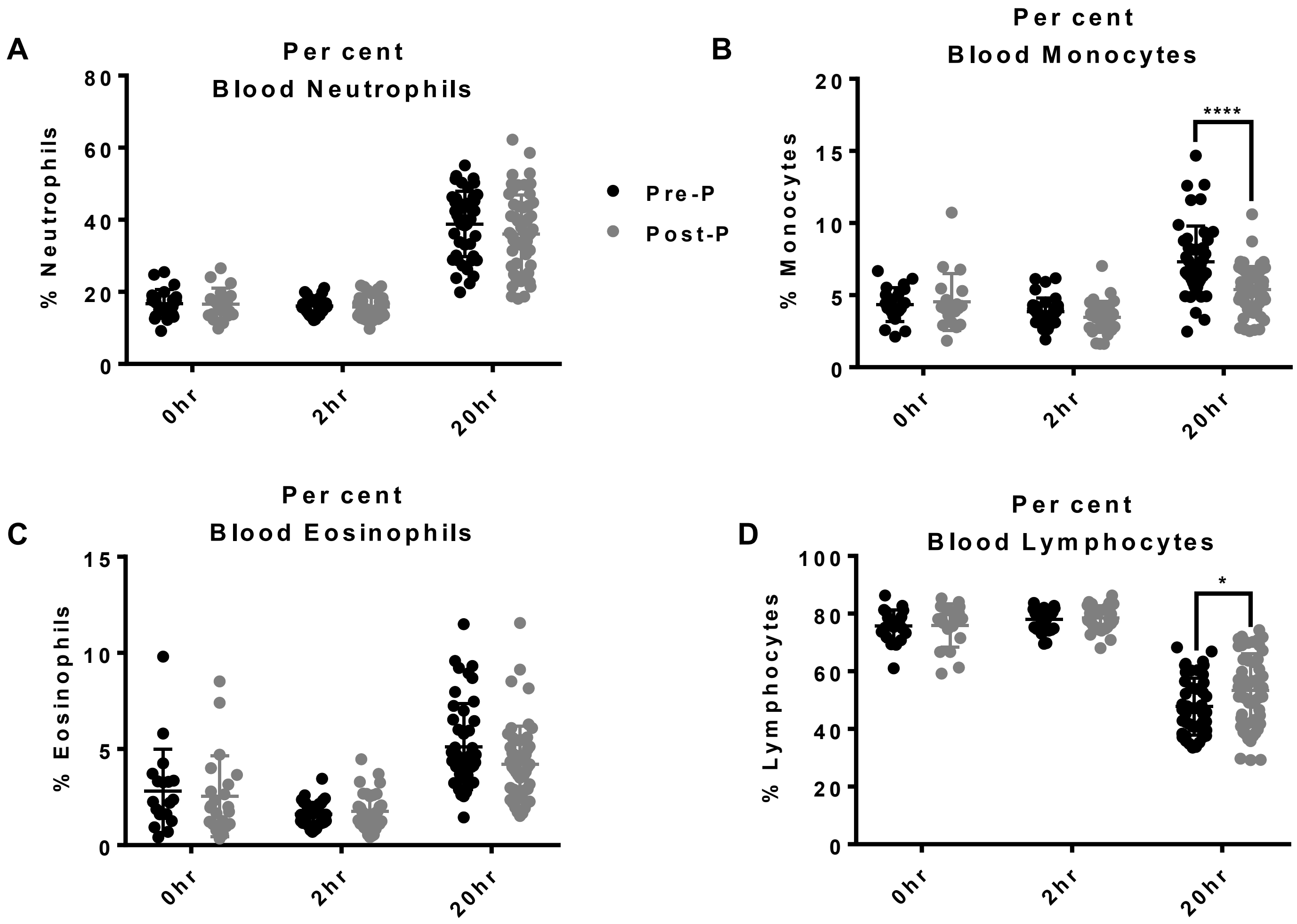

Figure S1. Pre and post-pubertal white blood cell composition during endotoxemia. All data for **(a)** % neutrophils, **(b)** % monocytes, **(c)** % eosinophils, and **(d)** % lymphocytes were obtained using the Hemavet 950 blood analyser. N≥19/group at 0hr, N≥35/group at 2hr, and N≥46/group at 20hr. Significant differences in per cent cell composition between pre- and post-pubertal mice are labelled with **** ($p<0.0001$), ** ($p<0.01$), or * ($p<0.05$). All comparisons were made using Two-way ANOVA followed by Tukey's multiple comparisons test.

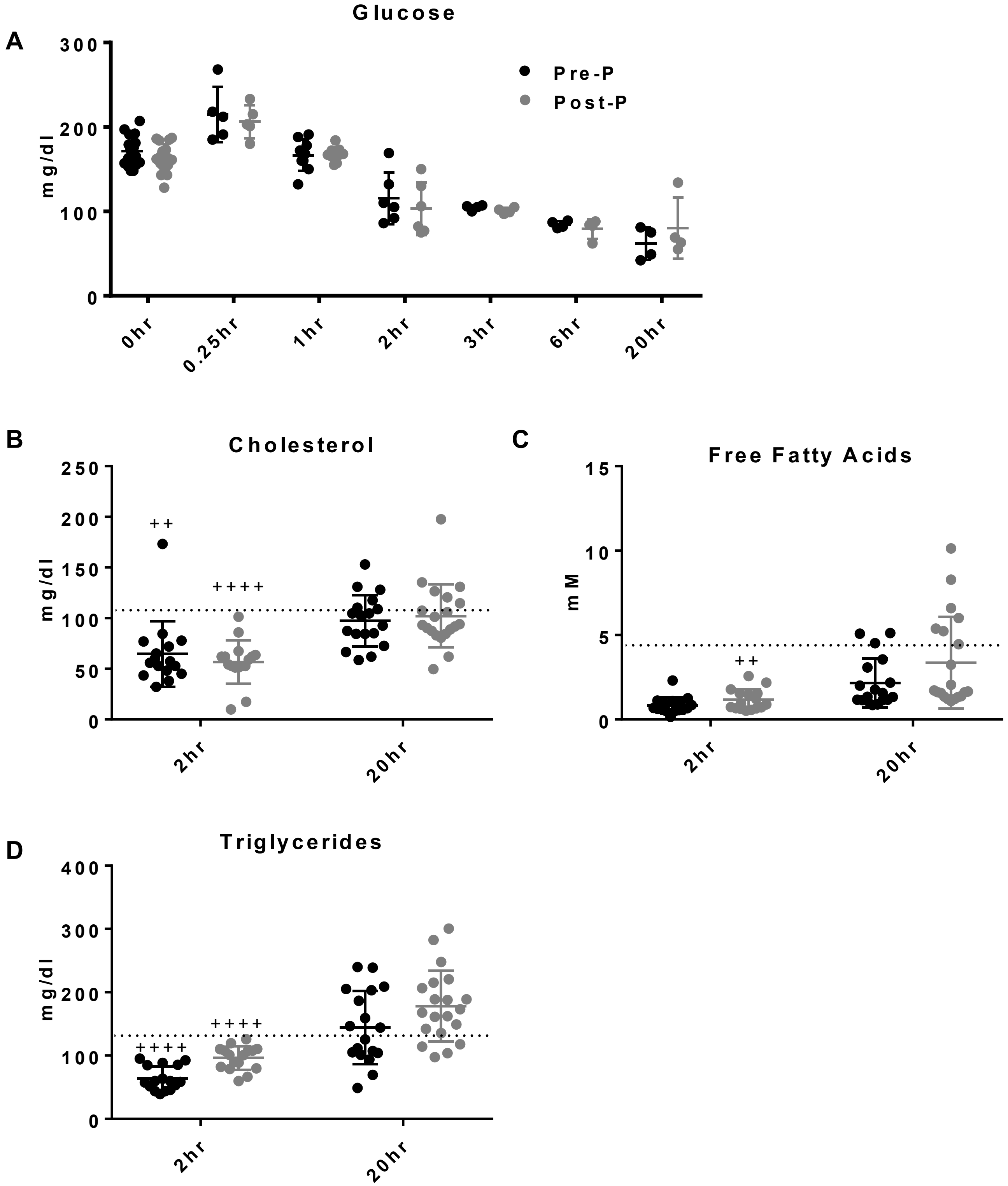

Figure S2. Serum metabolic factors are similar in endotoxemic pre- and post-pubertal mice. (a) Glucose was quantified in whole blood using the Easy Step Blood Glucose Monitoring System at multiple time points following LPS administration. For each time point, N≥4/group. **(b)** Cholesterol (CL), **(c)** Free Fatty Acids (FFA),

and **(d)** Triglycerides (TG) were quantified in serum samples isolated from pre- and post-pubertal mice at 2 and 20 hour time points. ($N \geq 16$/group.) The dotted line represents the mean value for each metabolic factor in normal serum. Time 0 values were similar across age groups and were pooled. Significant changes in the concentration of each metabolite over time (between 2 and 20 hours) in either pre- or post-pubertal mice are labelled with ++++ ($p<0.0001$) or ++ ($p<0.01$). All comparisons were made using Two-way ANOVA followed by Tukey's multiple comparisons test.

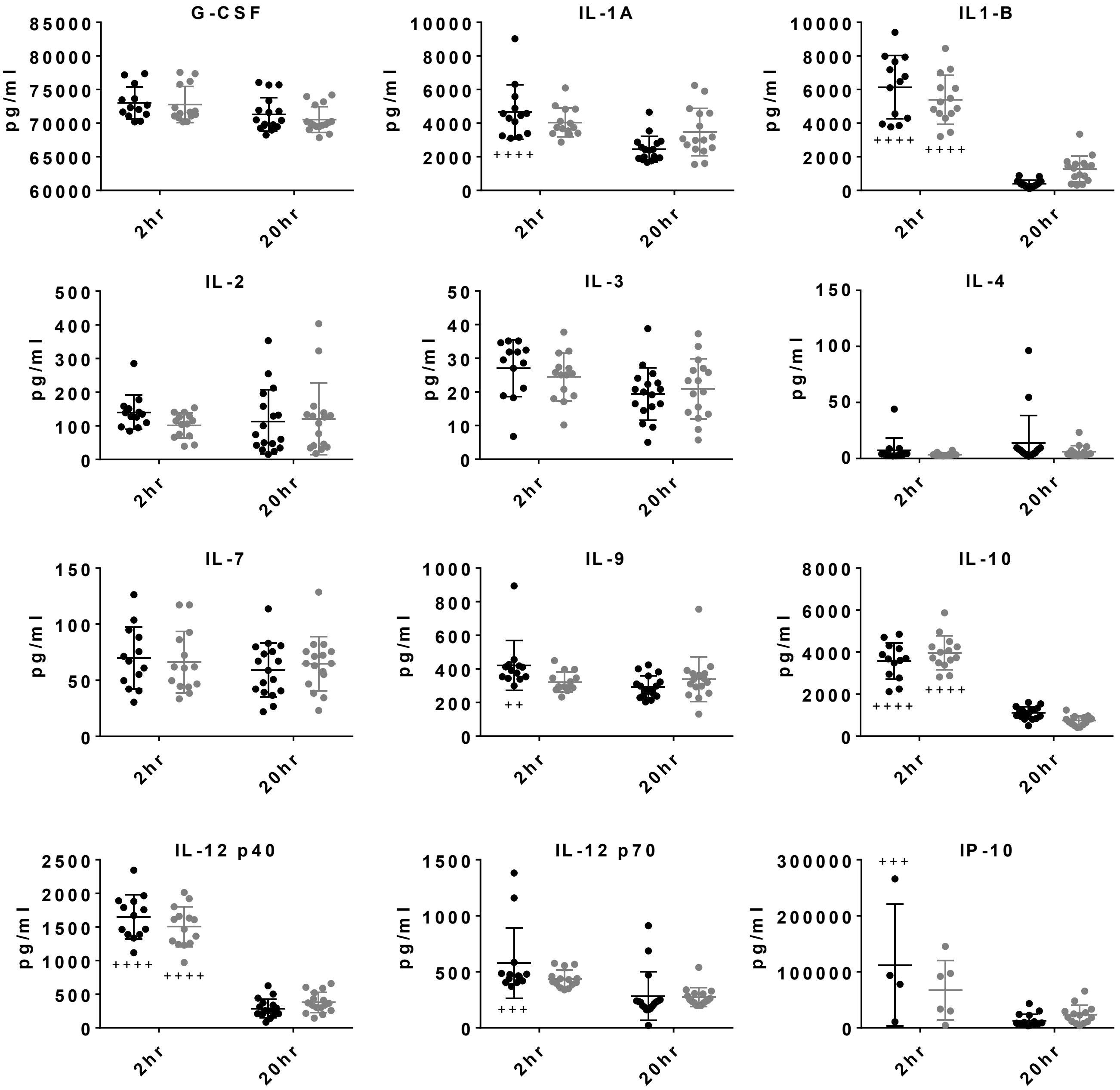
G-CSF
IL-1A
IL1-B
IL-2
IL-3
IL-4
IL-7
IL-9
IL-10
IL-12 p40
IL-12 p70
IP-10
pg/ml
2hr
20hr
++++
++
+++

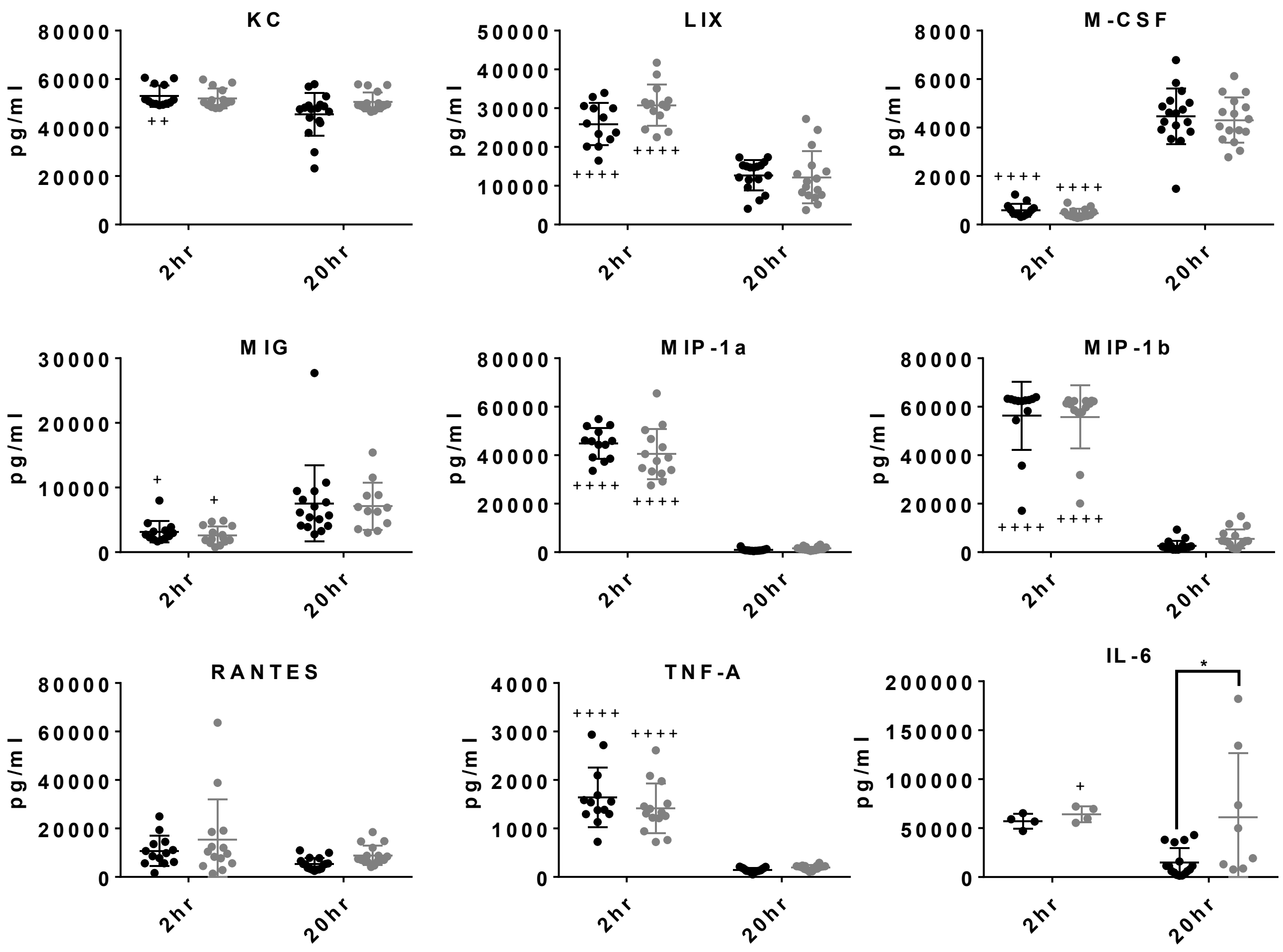

Figure S3. Serum cytokine expression following endotoxemia. Serum samples from pre- and post-pubertal C57Bl/6 mice were subjected to a 32-plex cytokine assay (N≥9/group). For all graphs, pre- and post-pubertal data are shown in black and grey respectively. Data points above or below the detectable limit of the assay were not included. Significant age-associated differences in cytokine concentration are labelled with * ($p<0.05$). Significant changes in concentration between 2 and 20 hours for either pre- or post-pubertal mice are labelled with ++++ ($p<0.0001$), +++ ($p<0.001$), ++ ($p<0.01$), or + ($p<0.05$). All comparisons were made using Two-way ANOVA followed by Tukey's test for multiple comparisons.

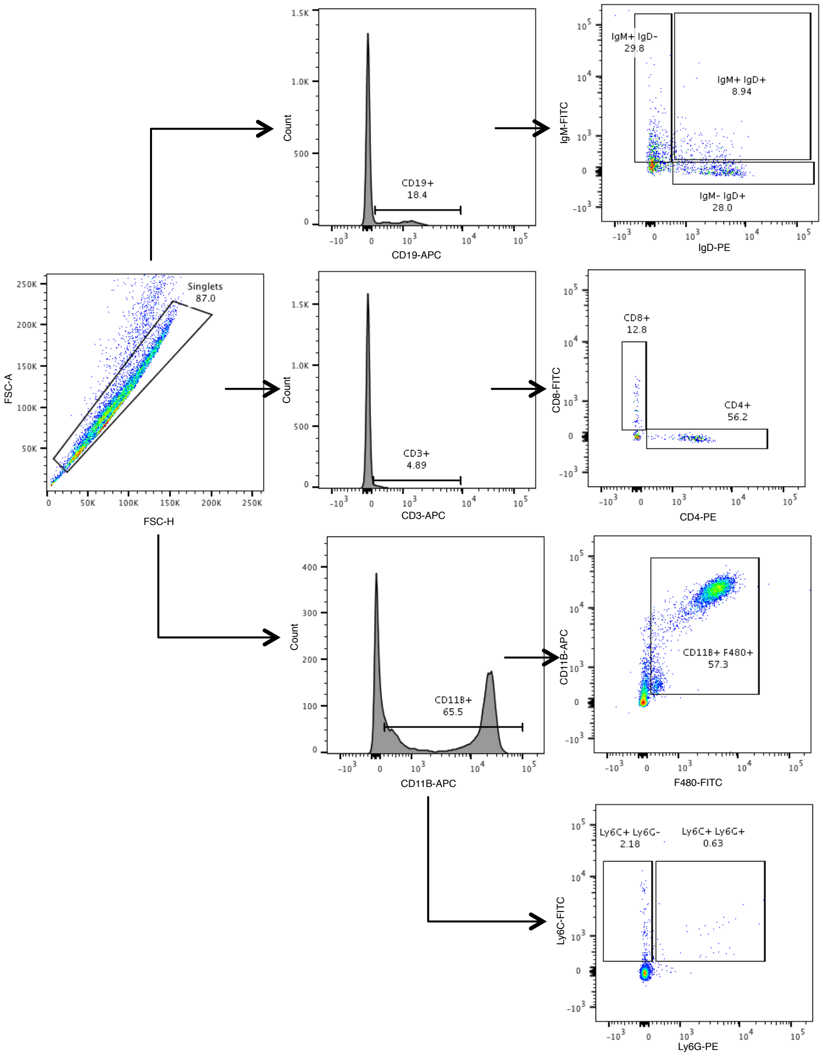

Figure S4. Flow cytometry experiment gating strategy and representative dot plots used for assessing endotoxin-induced changes to peritoneal cell composition. For experiments detailed in Figures 4 and S5 the above gating strategy was employed. Peritoneal cells were blocked to prevent non-specific binding and treated with combinations of antibodies for immuno-phenotypic analysis of the following: CD11B$^+$ myeloid cells with associated F480$^+$ macrophages, Ly6C$^+$LyG$^+$ neutrophilic cells and Ly6C$^+$Ly6G$^-$ monocytic cells; CD19$^+$ B Cells with associated IgM$^+$ and IgD$^+$ subsets, and CD3$^+$ T cells with CD4$^+$ and CD8$^+$ subsets. Ten thousand singlet cells were selected for analysis of cellular markers. The above pseudocolor plots were created using FlowJo and are representative of an individual post-pubertal mouse.

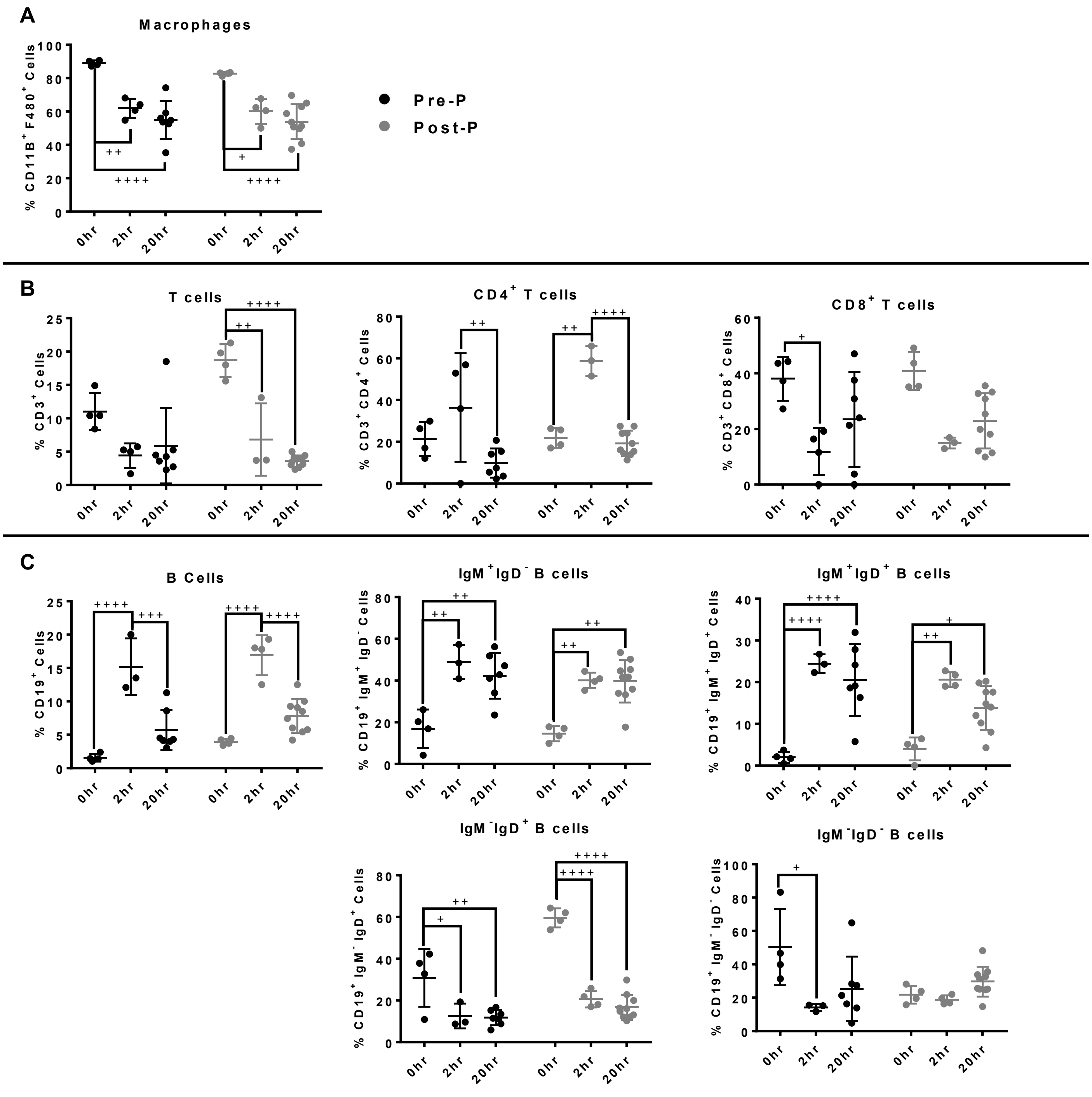

Figure S5. Changing peritoneal cell profiles in response to endotoxemia. At 2 and 20 hours following endotoxemia, peritoneal cells from pre- and post-pubertal mice were collected through peritoneal lavage. Cells were blocked to prevent non-specific binding and then treated with different combinations of antibodies for subsequent immuno-phenotypic analysis by flow cytometry. These include **(a)** $CD11B^{+}F480^{+}$ Macrophages, **(b)** $CD19^{+}$ B Cells with associated **(c)** IgM^{+} and IgD^{+} subsets, and **(d)** $CD3^{+}$ T cells with associated **(e)** $CD4^{+}$ and $CD8^{+}$ subsets. (N≥3/group). For each sample, a total of ten thousand cells were analysed using the gating

strategy found in Figure S4. Significant differences in concentration between pre- and post-pubertal mice are labelled with ** ($p<0.01$) or * ($p<0.05$). Significant changes in the per cent composition of each cell type over time in either pre- or post-pubertal mice are labelled with ++ ($p<0.01$) or + ($p<0.05$). All comparisons were made using Two-way ANOVA followed by Tukey's multiple comparisons test.

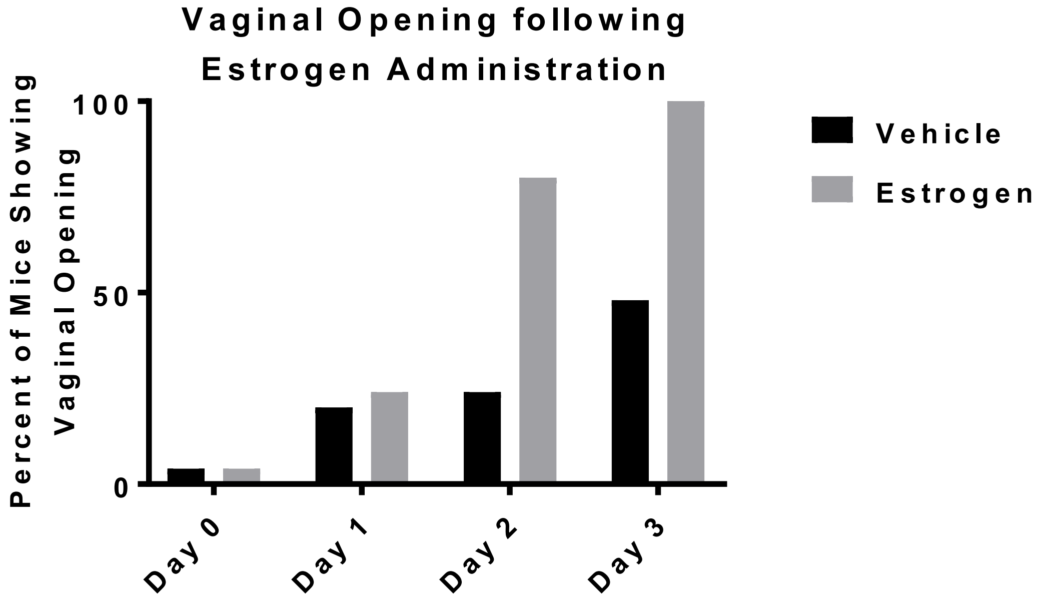

Figure S6. Vaginal opening following treatment with oestrogen or vehicle. Pre-pubertal CD-1 mice were pre-treated with daily subcutaneous injections of 17β-Oestradiol at 100µg/ml (or vehicle (0.4 % DMSO) suspended in corn oil for three days prior and once on the day of *E.coli* endotoxin injection. Vaginal opening was examined daily and recorded. Oestrogen-treated mice had 100 % vaginal opening by day 3 of oestrogen treatment. (N=25 /group; 2 experiments.)

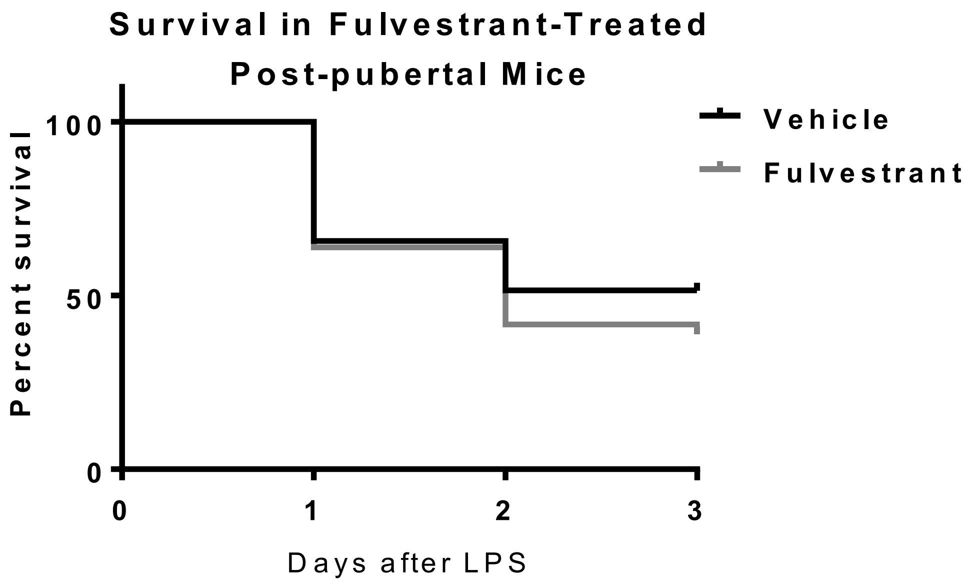

Figure S7. Effects of fulvestrant pre-treatment on endotoxemia survival. Post-pubertal CD-1 mice were treated with daily subcutaneous 100μl injections of fulvestrant (Sigma; I4409) at 2mg/ml or vehicle (0.8 % DMSO) in corn oil for three days prior and once on the day of endotoxin injection. N≥35/group; 2 experiments. Per cent survival was compared using a log rank Mantel Cox test.

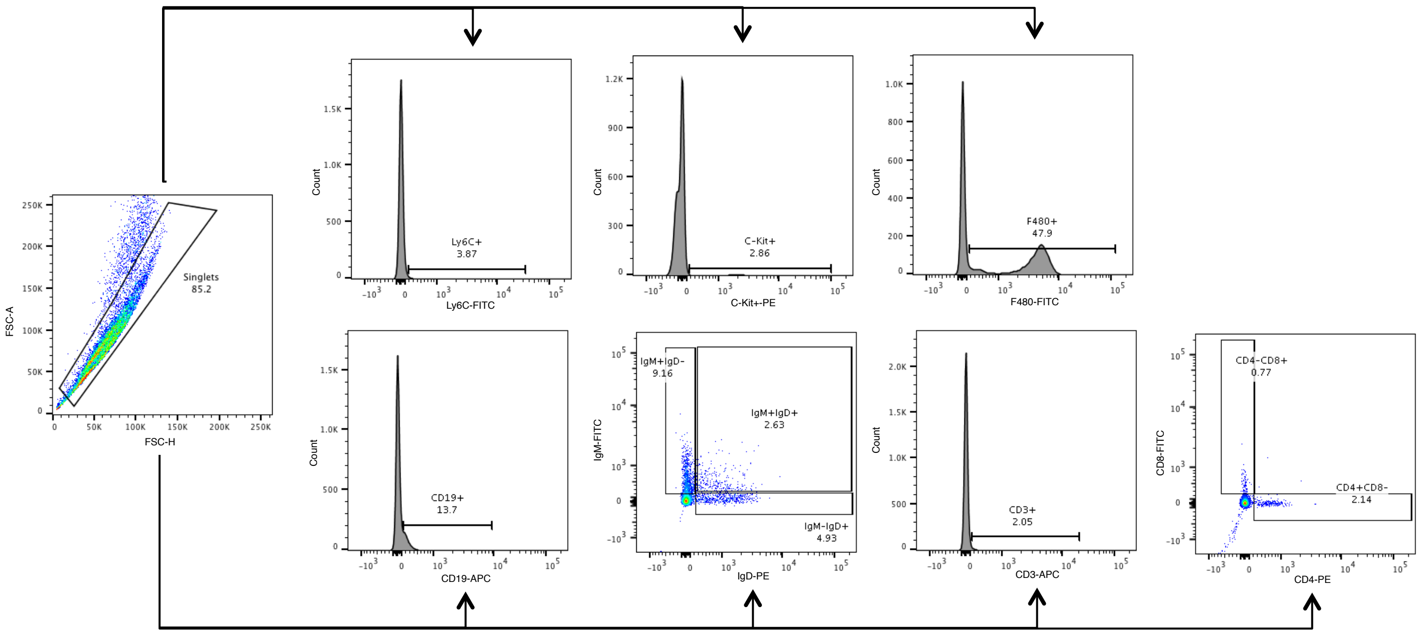

Figure S8. Flow cytometry experiment gating strategy and representative dot plots used for naïve peritoneal cell phenotyping. For experiments detailed in Figure 6 the above gating strategy was employed. Peritoneal cells were blocked to prevent non-specific binding and treated with combinations of antibodies for immuno-phenotypic analysis of the following: F480$^+$ macrophages, Ly6C$^+$ monocytes and neutrophils, C-kit$^+$ mast Cells, CD19$^+$ B Cells, IgM$^+$ and IgD$^+$ B cell subsets, and CD3$^+$ T cells, CD4$^+$ and CD8$^+$ T cell subsets. Ten thousand singlet cells were selected for analysis of cellular markers. The above pseudocolor plots were created using FlowJo and are representative of an individual post-pubertal mouse.

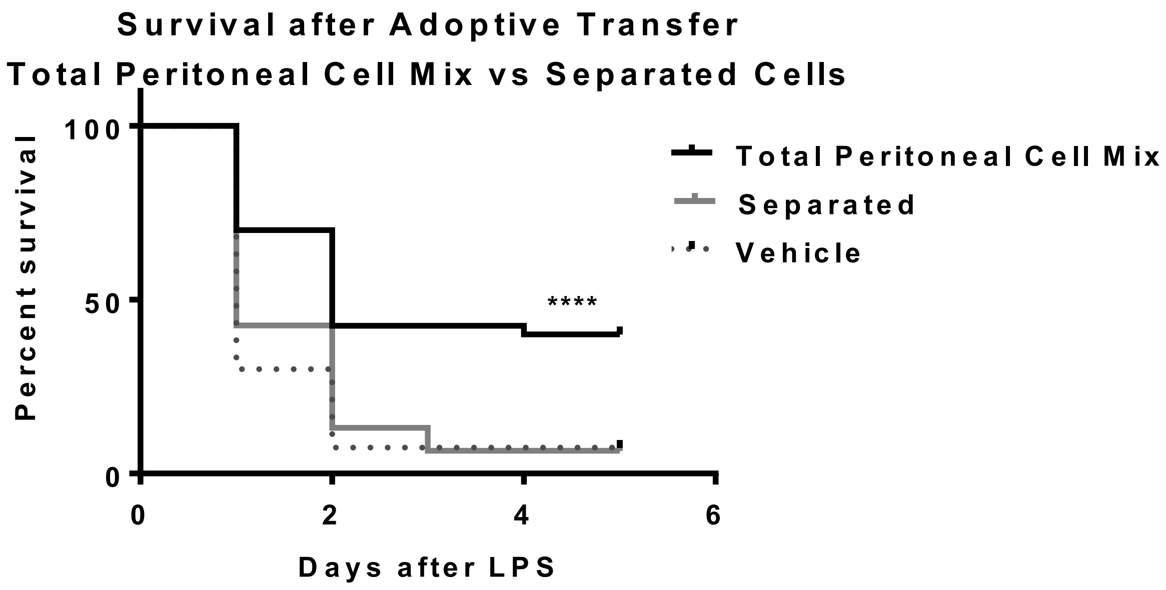

Figure S9. Adoptive transfer of pre-pubertal peritoneal cells separated using magnetic beads failed to improve survival. Naïve peritoneal cells were collected from pre-pubertal mice by peritoneal lavage and processed for adoptive transfer. Recipient post-pubertal mice were administered (i.p.) 1mL of the non-separated total peritoneal cell mix, 1mL of the vehicle, PBS, or 1mL of a suspension made up of specific cell types separated using the Miltenyi MACS® system. Following incubation of the donor cells within the peritoneal cavity, recipient mice were then subjected to endotoxemia. The figure above summarises findings generated through 5 experiments. While the total peritoneal cell mix and vehicle groups served as positive and negative controls throughout all the experiments, the separated group includes data from mice administered positively or negatively selected macrophages, flow through cells, as well as separated macrophages recombined with flow through. Despite multiple separation strategies, the process always interfered with the protective effect of adoptive transfer Differences in survival between mice administered the non-separated, total pre-pubertal peritoneal cell mix vs. mice receiving separated cells or vehicle were **** ($p<0.0001$). Per cent survival was compared using a log rank Mantel Cox test.
